# NosL is a dedicated copper chaperone for assembly of the Cu_Z_ center of nitrous oxide reductase†
†Electronic supplementary information (ESI) available. See DOI: 10.1039/c9sc01053j


**DOI:** 10.1039/c9sc01053j

**Published:** 2019-04-18

**Authors:** Sophie P. Bennett, Manuel J. Soriano-Laguna, Justin M. Bradley, Dimitri A. Svistunenko, David J. Richardson, Andrew J. Gates, Nick E. Le Brun

**Affiliations:** a Centre for Molecular and Structural Biochemistry , School of Chemistry , University of East Anglia , Norwich Research Park , Norwich , NR4 7TJ , UK . Email: n.le-brun@uea.ac.uk; b Centre for Molecular and Structural Biochemistry , School of Biological Sciences , University of East Anglia , Norwich Research Park , Norwich , NR4 7TJ , UK . Email: a.gates@uea.ac.uk; c School of Biological Sciences , University of Essex , Wivenhoe Park , Colchester CO4 3SQ , UK

## Abstract

Nitrous oxide reductase (N_2_OR) is the terminal enzyme of the denitrification pathway of soil bacteria that reduces the greenhouse gas nitrous oxide (N_2_O) to dinitrogen. In addition to a binuclear Cu_A_ site that functions in electron transfer, the active site of N_2_OR features a unique tetranuclear copper cluster bridged by inorganic sulfide, termed Cu_Z_. In copper-limited environments, N_2_OR fails to function, resulting in truncation of denitrification and rising levels of N_2_O released by cells to the atmosphere, presenting a major environmental challenge. Here we report studies of *nosL* from *Paracoccus denitrificans*, which is part of the *nos* gene cluster, and encodes a putative copper binding protein. A *Paracoccus denitrificans* Δ*nosL* mutant strain had no denitrification phenotype under copper-sufficient conditions but failed to reduce N_2_O under copper-limited conditions. N_2_OR isolated from Δ*nosL* cells was found to be deficient in copper and to exhibit attenuated activity. UV-visible absorbance spectroscopy revealed that bands due to the Cu_A_ center were unaffected, while those corresponding to the Cu_Z_ center were significantly reduced in intensity. *In vitro* studies of a soluble form of NosL without its predicted membrane anchor showed that it binds one Cu(i) ion per protein with attomolar affinity, but does not bind Cu(ii). Together, the data demonstrate that NosL is a copper-binding protein specifically required for assembly of the Cu_Z_ center of N_2_OR, and thus represents the first characterised assembly factor for the Cu_Z_ active site of this key environmental enzyme, which is globally responsible for the destruction of a potent greenhouse gas.

## Abstract 2

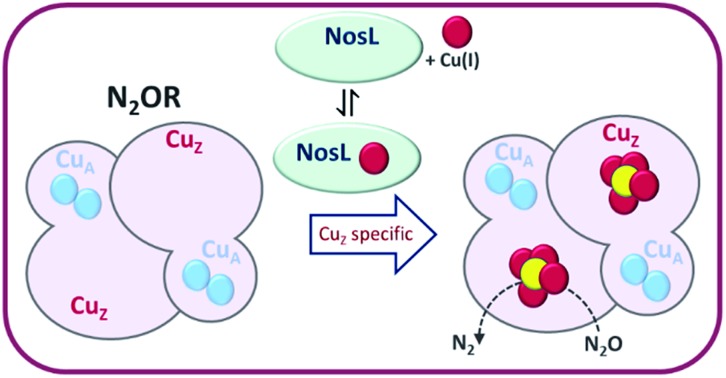
The Cu(i)-binding protein NosL functions specifically as an assembly factor for the unique Cu_Z_ centre of nitrous oxide reductase (N_2_OR).

## Introduction

Nitrous oxide (N_2_O) is a significant greenhouse gas with a ∼300 fold greater global warming potential than CO_2_ and an ability to deplete stratospheric ozone.^1^ Agriculture produces 65% (6.8 Tg N–N_2_O per year) of the total N_2_O emitted each year. The main contributor within this sector is the soil microbial community, which produces 40% of these emissions.^2^ The surge in atmospheric N_2_O from 270 ppb to 324 ppb over the last 100 years correlates strongly with the use of anthropogenic nitrogen-based fertilisers in farming to improve crop yield.^3^ The doubling of available nitrogen in the environment has enriched a class of soil dwelling microorganisms called denitrifiers, which respire anaerobically by reducing nitrate to dinitrogen (N_2_) gas *via* the free intermediates nitrite, nitric oxide and N_2_O using different metallo-enzymes. Environmental factors such as soil pH, moisture, carbon to nitrogen ratio, temperature and a lack of copper^4^–^6^ have all been identified as factors leading to increased N_2_O emissions from these microbes.

Encoded by the *nosZ* gene, the cupro-enzyme nitrous oxide reductase (N_2_OR or NosZ) catalyses the 2-electron reduction of N_2_O to N_2_. Two distinct and approximately equally abundant clades of N_2_OR-containing bacteria and archaea have been identified.^7^ Importantly, clade II members act as an N_2_O sink, while members of clade I, such as α-, β- and γ-proteobacteria, are able to produce and remove N_2_O under optimum conditions. In order to assist with future strategies for the control of emissions from soil ecosystems, a key task is to explore how the enzyme is produced and matured in N_2_O emitting bacteria.^3^

N_2_OR in the denitrifying α-proteobacterium *Paracoccus denitrificans* (*Pd*N_2_OR) is exported to the periplasm *via* the twin-arginine translocation (TAT) pathway,^8^,^9^ where its two copper centers, Cu_A_ and Cu_Z_, are assembled. Several crystal structures are now available for N_2_OR,^10^–^13^ revealing that the Cu_A_ site is housed in a C-terminal cupredoxin domain, while the Cu_Z_ site lies within the N-terminal seven bladed β-propeller domain. The Cu_A_ center contains two copper ions that are bridged by two conserved Cys residues and further coordinated by Met, His and Trp ligands to form a site that closely resembles the electron transfer Cu_A_ site present in subunit II of cytochrome *c* oxidase.^14^ In N_2_OR the Cu_A_ site also acts as an electron shuttle, accepting electrons from small electron donors such as cytochrome *c*_550_ (ref. 15) or pseudoazurin, in *P. denitrificans*,^16^ for the reduction of N_2_O at the Cu_Z_ site. This center comprises four copper atoms coordinated by seven conserved His residues and bridged by one ([4Cu:S]) or two ([4Cu:2S]) sulfides, depending on the presence or absence of O_2_, respectively, during purification.^10^,^12^

A major challenge that is particularly important for addressing N_2_O emissions from soil is to understand how the cofactor sites of N_2_OR are assembled and, in doing so, identify assembly/chaperone systems that are involved. For clade I organisms, including *P. denitrificans*, any such systems must be periplasmic, as mutations in the TAT leader sequence of N_2_OR results in the protein remaining in the cytoplasm, in a folded but copper-free state.^17^ Studies of the biogenesis of copper cofactor sites of eukaryotic cytochrome *c* oxidase have identified the proteins Cox17 and ScoB, which are involved in the assembly of the Cu_A_ site,^18^ and prokaryotic homologues PCu_A_C and SenC have been implicated in the maturation of the Cu_A_ site of the aa_3_-type cytochrome *c* oxidase from *Rhodobacter sphaeroides*.^19^

In comparison, little is known about the assembly of the Cu_Z_ site. The *nos* gene cluster of clade I N_2_O reducing bacteria vary between denitrifying phyla but contain, in addition to *nosZ*, five other core genes (*nosRZDFYL*), while predominantly α- and β-proteobacteria members also contain a further two genes (*nosC* and *nosX*). *nosDFY* are predicted to encode an ABC-type transporter homologous to ATM1 from eukaryotes, which transports a sulfur-containing species out of the mitochondrion for Fe–S cluster assembly in the cytoplasm,^20^ suggesting that these proteins are likely involved in supplying sulfide for assembly of the Cu_Z_ center. In *P. denitrificans*, downstream of *nosDFY* are two further accessory genes that are part of the same operon, *nosL* and *nosX*. The only mutation analysis of *nosL* has been in *P. stutzeri*^9^ and of *nosX* in *P. denitrificans*,^21^ both of which led to the conclusion they alone are not important for whole-cell N_2_O reduction. However, *nosL* is part of the denitrification core gene cluster, its expression is responsive to cellular copper status,^22^ and the NosL protein has been shown to bind copper,^23^ suggesting that it plays a role in maturation or activation of N_2_OR.

Here, we report genetic and biochemical studies of *P. denitrificans nosL*/NosL. The data demonstrate that NosL is a Cu(i)-binding protein that is required for efficient assembly of the N_2_OR Cu_Z_ center, and thus represents the first characterised assembly factor for this unique metal center in biology.

## Results

### NosL is required for N_2_OR activity under copper-limited conditions

**Fig. 1:**
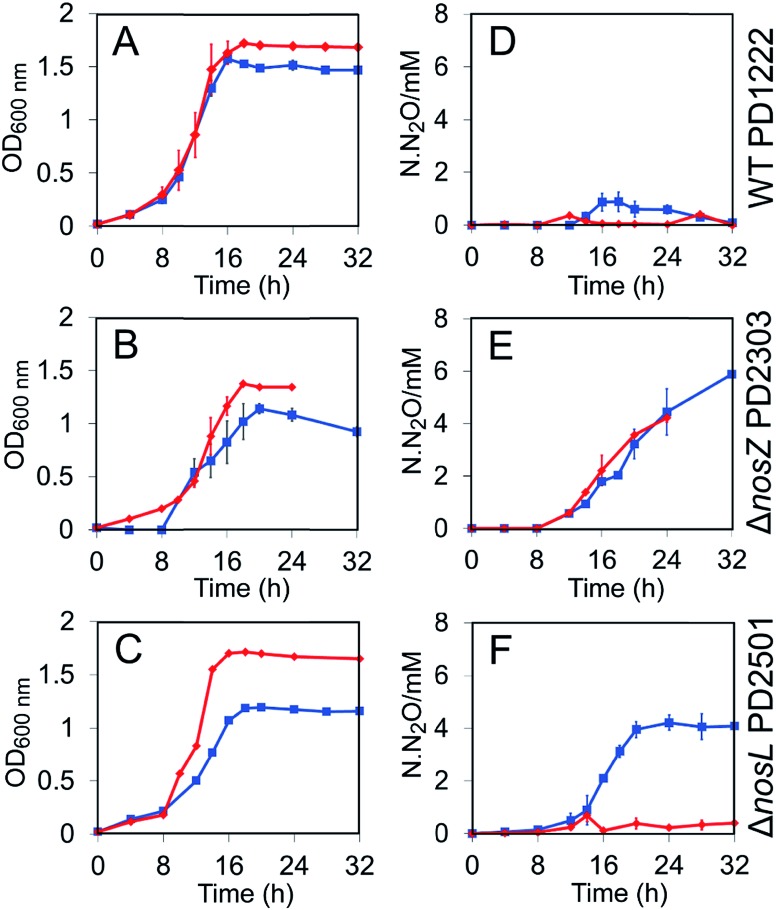
Growth and N_2_O emission profiles for *P. denitrificans* WT and mutant strain cultures. (A)–(C) OD_600 nm_ as a function of time for WT PD1222 (A), Δ*nosZ* deletion mutant PD2303 (B), and Δ*nosL* deletion mutant PD2501 (C). (D)–(F) N_2_O emissions as N·N_2_O (millimolar N in the form of N_2_O) for PD1222 (D), PD2303 (E), and PD2501 (F). Strains were grown in anaerobic batch culture conditions in Cu-sufficient media (red diamonds) and Cu-limited media (blue squares). Bars represent SE.

Wild type PD1222 (WT), Δ*nosZ* (lacking the gene encoding N_2_OR) and Δ*nosL* strains (Table S1†) were cultured under both Cu-sufficient and Cu-limited anaerobic conditions and their growth characteristics investigated. For the WT strain, growth yield was affected under Cu-limited conditions (Fig. 1A). For the Δ*nosZ* strain, growth rate and yield were affected under both conditions, consistent with the importance of N_2_OR for optimal growth under anaerobic nitrate-sufficient conditions (Fig. 1B). Growth of the Δ*nosL* strain was similar to WT under Cu-sufficient conditions, but was attenuated under Cu-limited conditions, exhibiting a maximum OD_600 nm_ similar to the Δ*nosZ* strain (Fig. 1C).

The impact of *nosL* deletion on N_2_OR activity *in vivo* was assessed by measuring N_2_O levels in the headspace of cultures. Consistent with previous studies, no N_2_O was accumulated by the WT strain under Cu-sufficient conditions and only a transient low level of N_2_O (<1 mM at ∼16 h) was observed under Cu-limited conditions (Fig. 1D), where the latter is likely due to lower transcription of *nosZ* under these conditions.^22^ In contrast, the Δ*nosZ* mutant exhibited a marked Nos-negative phenotype (Nos^–^) regardless of copper levels, with N_2_O emitted from cultures to >4 mM after 24 h (Fig. 1E). Like the WT, essentially no N_2_O was emitted from Δ*nosL* cultures under Cu-sufficient conditions. Importantly, however, after 24 h of growth and once the cells had reached stationary phase, up to 4 mM N_2_O was detected in the headspace of Δ*nosL* cultures under Cu-limited conditions, similar to the Δ*nosZ* strain (Fig. 1F).

The Nos^–^ phenotype of the Δ*nosL* strain under Cu-limited conditions was almost fully complemented by expression of a plasmid-borne functional *nosL* gene copy from a taurine inducible promoter (Fig. S1†). In particular, the extent of N_2_O release from the complemented Δ*nosL* mutant closely resembled the WT strain in the transient accumulation of N_2_O at ∼16–20 h.

### Absence of NosL results in a copper- and catalytically-deficient N_2_OR

To investigate the functional properties of NosL in relation to N_2_OR biogenesis, N_2_OR (NosZ) with a C-terminal Strep-II tag was overproduced in *P. denitrificans* strains grown under Cu-sufficient conditions and purified. N_2_OR from Δ*nosL* cells contained on average 4 Cu atoms per monomer, compared to ∼6 for the enzyme from Δ*nosZ* cells (Table 1). N_2_OR from a Δ*nosZL* double mutant contained ∼3.5 copper atoms per monomer, demonstrating that the presence of the chromosomal *nosZ* gene in the Δ*nosL* cells had little effect on the copper content of the tagged N_2_OR (NosZ) (Table 1).

**Table 1:** Copper content and activities of N_2_OR enzymes from different *P. denitrificans* strains

Strain	Cu-sufficient		Cu-limited
	Cu/monomer^*a*^	Maximum specific activity^*b*^ (μmol N_2_O min^–1^ mg^–1^)	Cu/monomer
Δ*nosL*/pMSL002 (StrepII tagged-N_2_OR)	4.1 ± 0.43	95.7 ± 3.9	N.D.
Δ*nosZ*/pMSL002	5.9 ± 0.56	196.2 ± 9	4.8 ± 0.42
Δ*nosZL*/pMSL002	3.4 ± 0.26	93.6 ± 0.8	N.D.

^*a*^Total copper contents per monomer were determined using a BCS copper assay.

^*b*^N_2_O reductase activity was determined using a reduced methyl viologen assay (μmol N_2_O min^–1^ mg^–1^ enzyme). Proteins were pre-incubated with a 500-fold excess reduced methyl viologen for 150 min prior to activity assay. All reactions were carried out in triplicate and SD is shown. N.D., not detectable.

As purification of N_2_OR was carried out aerobically, the enzyme from Δ*nosZ* cells contained the 
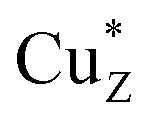
 (pink, form II) form of the active site, which is catalytically inactive.^24^ To activate the isolated N_2_OR to the fully reduced form, the enzyme was incubated with excess reduced methyl viologen at room temperature^25^,^26^ for 150 min, at which point N_2_O reductase activity had reached a maximum. N_2_OR from Δ*nosL* and Δ*nosZL* cells exhibited significantly lower maximum activities than N_2_OR from Δ*nosZ*, see Table 1.

### Absence of NosL results in N_2_OR deficient in the Cu_Z_ center

**Fig. 2:**
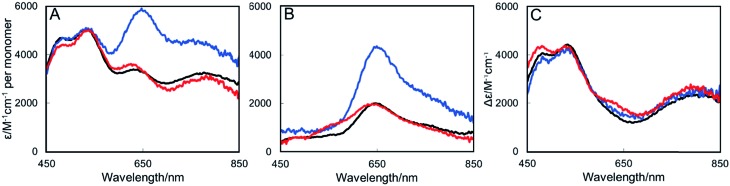
UV-Visible absorbance spectra of N_2_OR purified from Cu-sufficient *P. denitrificans* mutant strains. (A) N_2_OR isolated from Δ*nosZ* PD2303 (blue line), Δ*nosL* PD2501 (black line) and Δ*nosZL* PD2505 (red line) following oxidation using ferricyanide. (B) As in (A) but following reduction using sodium dithionite. (C) Oxidized minus reduced difference spectrum. Oxidized spectra were normalised to *ε*_580 nm_ 5000 M^–1^ cm^–1^ per monomer as described by Rasmussen *et al.*^27^ Sample buffer contained 20 mM HEPES, 150 mM NaCl, pH 7.2.

The UV-visible absorbance spectrum of as isolated air-oxidized (pink, form II) strep-tagged N_2_OR purified from Δ*nosZ P. denitrificans* displayed bands at 480, 535 and 645 nm (Fig. 2A and S2†), in agreement with literature for N_2_OR enzymes from a range of bacteria.^11^,^26^–^29^ These features arise from S^2–^ to Cu(ii) charge-transfer transitions and transitions due to interactions between Cu(i) and Cu(ii) ions.^27^ The bands at 480 and 550 nm correspond to the Cu_A_ centre, while that at 645 nm is characteristic of the Cu_Z_ centre (in its 
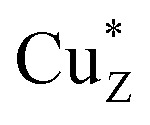
 form).^29^

The spectra of N_2_OR enzymes purified from Δ*nosL* and Δ*nosZL* strains were very similar in the 450–550 nm region, but exhibited significantly reduced intensity beyond 550 nm (Fig. 2A). The apparent absorbance maximum was shifted to ∼635 nm, consistent with the enhanced relative influence of the underlying absorbance due to Cu_A_ (maximum at 535 nm). Reduction with sodium dithionite resulted in the spectra shown in Fig. 2B. Bands at 480 and 540 nm were lost, consistent with the reduction of the Cu_A_ center to its colorless diamagnetic Cu(i)/Cu(i) state. The remaining band is characteristic of the Cu_Z_ center following addition of dithionite.^27^ N_2_OR from both Δ*nosL* and Δ*nosZL* strains exhibited a much less intense Cu_Z_ absorbance than that from Δ*nosZ*, indicating diverse occupancies of the center. Ferricyanide-oxidized minus dithionite-reduced difference spectra (Fig. 2C) closely overlay, particularly in the 450–550 nm region, demonstrating that the N_2_OR Cu_A_ centers of the different enzymes are essentially identical, and close to fully populated, as estimated by the measured extinction coefficients.^27^ Together, these data demonstrate that N_2_OR isolated from a Δ*nosL* background is specifically deficient in its Cu_Z_ center.

### Absence of NosL under copper-limited growth conditions results in complete absence of Cu_A_ and Cu_Z_ centers in N_2_OR

**Fig. 3:**
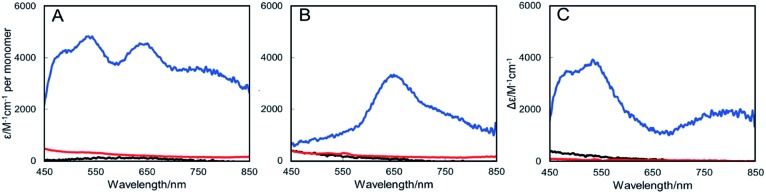
UV-Visible absorbance spectra of N_2_OR purified from Cu-limited *P. denitrificans* mutant strains. (A) N_2_OR isolated from Δ*nosZ* PD2303 (blue line), Δ*nosL* PD2501 (black line) and Δ*nosZL* PD2505 (red line) following oxidation using ferricyanide. (B) As in (A) but following reduction with sodium dithionite. (C). Oxidised minus reduced difference spectra. Spectra were normalised to the protein concentration. Sample buffer contained 20 mM HEPES, 150 mM NaCl, pH 7.2.

The copper determinations, activity assays and spectroscopic characterizations of N_2_OR described above were performed with samples isolated from cultures grown in copper-sufficient media, in which only a minor growth phenotype for the Δ*nosL* strain was observed (Fig. 1). Thus, N_2_OR characteristics under low copper, where N_2_O is generated from cultures, were investigated. N_2_OR from Δ*nosZ* cells contained ∼5 Cu per monomer (Table 1) with an absorbance spectrum indicative of complete, or near complete, Cu_A_ center population, but a less than stoichiometric population of Cu_Z_ (Fig. 3). In contrast, N_2_OR from Δ*nosL* and Δ*nosZL* cells contained no detectable copper (Table 1), and gave UV-visible absorbance spectra with no absorbance in the visible region (Fig. 3), indicating complete failure to assemble either of the copper centers of N_2_OR in the absence of NosL.

### NosL binds Cu(i) with attomolar affinity

NosL contains a Type-II signal peptidase recognition sequence that, when cleaved, produces a protein with an N-terminal Cys residue that is predicted to bind lipid and anchor NosL into the outer membrane.^23^,^30^ The NMR solution structure of NosL, lacking its membrane anchor sequence, from the β-proteobacterium *Achromobacter cycloclastes* revealed two independent homologous domains with an unusual ββαβ topology.^31^ The same authors showed that the protein binds Cu(i) specifically and XAFS data were consistent with a Cu(i) coordination consisting of S and N/O ligands.^23^ To determine the biochemical/biophysical properties of *P. denitrificans* NosL, the protein lacking its periplasmic export signal sequence and its predicted N-terminal Cys residue^23^ was purified resulting in a metal-free form of the protein, which gave a mass of 18 890 Da (predicted mass 18 891 Da) by LC-ESI-MS (Fig. S3†). The final, gel filtration step of purification resulted in a broad elution band that suggested a mixture of monomer/dimer association states for NosL, a result confirmed by native PAGE (Fig. S4†), which showed two species of NosL. Similar observations were made for apo-NosL from *A. cycloclastes*.^23^

**Fig. 4:**
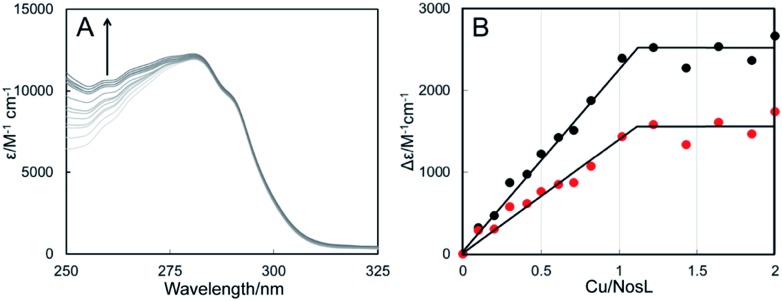
Copper-binding to NosL. (A) UV-visible absorbance titration of NosL (21 μM) with Cu(i). (B) Plots of Δ*ε*_260 nm_ (black circles) and Δ*ε*_265 nm_ (red circles) as a function of Cu(i) ions per protein molecule. NosL was in 100 mM MOPS, 100 mM NaCl, pH 7.5.

Titration of apo-NosL with Cu(i) resulted in the series of spectra shown in Fig. 4A, in which broad absorbance in the near-UV region of the spectrum was observed to gradually increase and saturate at a level of 1 Cu(i) per NosL (Fig. 4B). The absorbance is characteristic of charge transfer transitions involving Cu(i) coordinated to a cysteine thiolate.^32^ Cu(i)-binding was also investigated by CD spectroscopy, which confirmed the tight association of one Cu(i) per protein but also suggested that further Cu(i) can associate with NosL, albeit weakly (Fig. S5†). Gel filtration and native PAGE analysis of Cu(i)-NosL (Fig. S4†) also demonstrated that Cu(i) binding does not significantly affect the association state equilibrium.

Equivalent titrations with Cu(ii) followed by absorbance spectroscopy resulted in spectra very similar to those for Cu(i) (Fig. S6†), suggesting that Cu(ii) may undergo auto-reduction upon binding to NosL. This possibility was investigated using EPR (Fig. S7 and Table S2†). Addition of Cu(ii) to NosL resulted in a characteristic *S* = 1/2 Cu(ii) signal that, by comparison with a Cu(ii) standard, corresponded to only ∼8% of the Cu(ii) initially added. The same sample in the presence of EDTA (a Cu(ii) chelator), which prevents Cu(ii) ions in solution dimerizing to form EPR-inactive species, resulted in a Cu(ii) concentration of ∼11%. Similar experiments but with the addition of Cu(i) in place of Cu(ii) resulted in 4% and 8% in the absence and presence of EDTA, respectively (Table S2†). As isolated NosL contained <1% Cu(ii). Together, these data indicate that Cu(ii) undergoes auto-reduction to EPR silent Cu(i) upon binding to NosL.

**Fig. 5:**
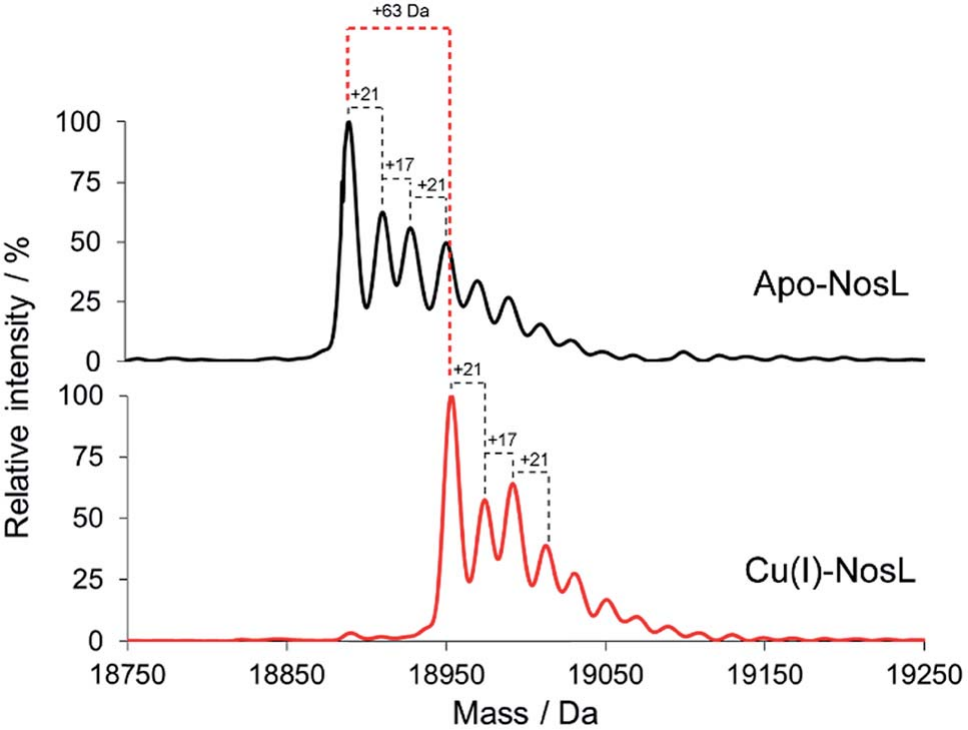
Deconvoluted ESI-MS of NosL (20 μM) in the apo-form and containing 1 Cu(i) per protein, as indicated, in 50 mM ammonium acetate, pH 7.8.

To further characterise Cu(i)-binding to NosL, ESI-MS under non-denaturing conditions, where non-covalent interactions are preserved, was also employed. Fig. 5 shows the deconvoluted spectrum of apo-NosL with the major peak at 18 890 Da (as observed by LC-ESI-MS, Fig. S3†), along with a number of lower intensity peaks to the higher mass side, due to non-covalent sodium and ammonium adducts. Attempts to remove these adducts from the non-denaturing MS, *via* buffer exchange, changes in pH and ionic strength, were unsuccessful. NosL was loaded with a single Cu(i) ion and the peak envelope of the resulting deconvoluted mass spectrum was at +63 Da relative to that of the apo-NosL spectrum (Fig. 5), consistent with the binding of a single Cu(i) ion.

Competition binding experiments using the high-affinity chelator BCS were used to determine the dissociation constant for Cu(i)-binding to NosL. Cu(i)-NosL was titrated with BCS, and the partition of Cu(i) between NosL and BCS determined from measured *A*_483 nm_, due to [Cu(BCS)_2_]^3–^, together with the well-characterised formation constant for Cu(BCS_2_)^3–^, *β*_2_ = 10^19.8^ (Fig. S8†)^33^. From these, an average *K*_d_ value of ∼4 × 10^–18^ M was determined^34^ (Table S3†), demonstrating very tight binding of Cu(i) to NosL.

## Discussion

Despite *nosL* being a core component of the *nos* gene clusters in a range of microorganisms, mutational studies^9^ have so far failed to reveal a function in N_2_O reduction. A *nosL* mutant of *Pseudomonas stutzeri* (containing an insertion towards the 3′ end of the gene) exhibited a slightly lower growth rate but produced active, holo-N_2_OR.^9^ The presence of a CXXC motif in the encoded protein prompted the suggestion that NosL might be a protein disulfide isomerase, but analysis of the sequence of NosL from *Sinorhizobium meliloti*^35^ showed that one of the Cys residues of the *P. stutzeri* protein is not conserved.

Here, a *nosL* deletion mutant of *P. denitrificans* was generated and compared to the WT and *nosZ* deletion strains, as growth and N_2_O benchmarks, under both Cu-limited and Cu-sufficient conditions. The *nosL* mutant exhibited a Cu-limited growth phenotype relative to WT, associated with a deficiency in the activity of N_2_OR, leading to accumulation of N_2_O in the culture headspace. The phenotype was complemented *in trans* by *nosL* and also under copper replete conditions, strongly suggesting that NosL functions in an aspect of copper metabolism. The reason why the previously reported *P. stutzeri nosL* mutant did not present a phenotype is unclear; one possibility is that there was sufficient copper in the growth medium to mask the phenotype, but growth conditions were not reported.^9^

Strep-tagged N_2_OR enzymes purified from Δ*nosZ*, Δ*nosL* and Δ*nosZL* mutants exhibited clear differences. Under Cu-sufficient conditions, N_2_OR from Δ*nosL* cells contained only ∼4 Cu per N_2_OR monomer and was substantially less active (though it was apparently sufficiently active *in vivo* to mask any obvious *in vivo* phenotype). Importantly, spectroscopic characterisation revealed that the N_2_OR protein from Δ*nosL* was specifically deficient in its Cu_Z_ center, demonstrating that NosL functions in the assembly of this unique biological metal center required for N_2_O destruction.

Under Cu-limited conditions, tagged N_2_OR purified from the Δ*nosZ* mutant contained nearly 5 Cu/monomer, while those from Δ*nosL* or Δ*nosZL* mutant strains lacked copper entirely, indicating complete failure to assemble either N_2_OR copper center in the absence of NosL. Thus, although the Cu_A_ can be reconstituted using copper alone,^36^ when copper is limited, NosL may also supply Cu for incorporation into Cu_A_.

The N-terminal sequence of NosL contains a periplasmic export signal and a lipobox that is predicted to be processed by Lgt, Lsp and Lnt enzymes, resulting in a mature protein that is membrane-anchored *via* a triacylated N-terminal Cys residue (indicated by a yellow arrowhead in Fig. S9†).^37^ This is likely a substrate of the Lol system,^37^ such that it is located in the outer membrane, with its soluble domains facing the periplasm. Studies of a soluble form of NosL, lacking its periplasmic targeting sequence and the N-terminal Cys residue that is acylated, revealed that it binds Cu(i) with attomolar affinity, a characteristic of many copper chaperones.

Our data are consistent with Cys coordination, in agreement with previous studies of *A. cycloclastes* NosL using thiol-specific reagents and EXAFS, with data from the latter consistent with a three or four coordinate Cu(i) center. The best fit was obtained for three coordinate Cu(i) with (O/N)S_2_ ligands.^23^ Alignment of NosL sequences from the two clades of N_2_O reducing bacteria identifies two conserved Cys residues: one through which the protein is believed to be anchored to the membrane (see, Fig. S9,† yellow arrowhead); and, one within a CXM motif that is likely to participate in Cu(i)-binding.^23^ The Met residue of this motif is also strictly conserved and was proposed to provide the second sulfur ligand identified by EXAFS.^23^ There is one other absolutely conserved Met residue but, as acknowledged by McGuirl *et al.*, there is no absolutely conserved His residue (Fig. S9†).

Separation of NosL proteins according to the clade to which the organism belongs may shed some further light on this, as it reveals residues that are conserved within, but not between, clades. Clade I NosL proteins, which include *P. denitrificans* and *A. cycloclastes*, contain a well-conserved His residue close to the CXM motif, resulting in a CXMX_3_H motif. Clade II NosL proteins do not contain this His but instead contain a conserved Cys residue at the N-terminal side of the CXM motif (resulting in a CXXCXM motif). *P. stutzeri* NosL is the exception to this, as it comes from a Clade I organism but is more similar to Clade II NosL proteins.

In summary, we identify here the first Cu chaperone with a specific role in Cu_Z_ center assembly. Our data indicate that NosL, which binds Cu(i) with attomolar affinity, is a key part of a high-affinity Cu trafficking pathway that enables assembly of the Cu_Z_ center of N_2_OR. The pathway functions under Cu-sufficient conditions, but becomes essential under Cu-limited conditions. During copper limitation, the NosL pathway may also play a key role in supplying Cu for the Cu_A_ center. How NosL delivers Cu to N_2_OR is unknown; it is likely that NosL functions directly in the transfer of Cu(i) to N_2_OR, and is thus a copper chaperone, but further studies are now needed to investigate this. The identification of NosL as a key component of a Cu-trafficking pathway for the assembly of the active holoreductase N_2_OR, the sole pivotal enzyme for N_2_O destruction, is a significant advance towards the long-term aim of mitigating microbial emissions of N_2_O into the atmosphere.

## Materials and methods

### Construction of mutant *nosL* and *nosZL* deficient strains of *P. denitrificans*

Unmarked deletions of *nosL* in *P. denitrificans* wild type or Δ*nosZ* backgrounds (Table S1†) were produced by the method of allelic replacement.^22^ Briefly, regions flanking *nosL* (Table S4†) were cloned into pK18*mobsacB* using *Eco*RI and *Pst*I sites to generate a suicide plasmid (pSPBN1, Table S1†) that was subsequently conjugated into *P. denitrificans* PD1222 or Δ*nosZ* PD2303 using the helper *E. coli* pRK2013 strain. Single cross-over recombination events were screened using spectinomycin (25 μg ml^–1^) and kanamycin (50 μg ml^–1^). Primary Spec^R^/Km^R^ transconjugants were grown to stationary phase in Luria Bertani broth (LB) with no antibiotic. Double cross over events were selected for using a high-salt modified LB agar supplemented with 6% (w/v) sucrose. Sucrose resistant colonies with Spec^R^ were screened using colony PCR and gene deletion confirmed by sequencing. Deletion strains were named PD2501 (Δ*nosL*) and PD2505 (Δ*nosZL*). For complementation of Δ*nosL* cells the coding sequence of Pden_4215 was synthesised (Genscript) with flanking 5′ *Nde*I and 3′ *Eco*RI restriction sites and sub-cloned into a taurine-inducible modified pLMB509 plasmid with gentamicin resistance (20 μg ml^–1^) to generate pSPBN2. The complementation plasmid was conjugated into the mutant strain using the helper *E. coli* pRK2013 strain and successful conjugants were Spec^R^/Gm^R^. Expression of *nosL* from the plasmid was induced by adding 1 mM taurine to the medium at the start of the growth experiment.

### Growth and phenotype analysis of cultures

Anaerobic minimal media batch cultures (400 ml) were grown in sealed Duran flasks (500 ml total volume), fitted with a septum to allow for gas-tight sample extraction. Minimal media consisted of: 30 mM succinate, 20 mM nitrate, 11 mM dihydrogen orthophosphate, 29 mM di-sodium orthophosphate, 0.4 mM magnesium sulfate, 1 mM ammonium chloride, pH 7.5. The minimal media was supplemented with a 2 ml l^–1^ Vishniac and Santer trace element solution^38^ where copper sulfate was present (Cu-sufficient, 12.8 μM) or excluded from the original recipe (Cu-limited, <0.5 μM), as previously described.^22^ Media were inoculated using a 1% inoculum from a starter culture to give a starting OD_600 nm_ of 0.02 and incubated at 30 °C. Samples of the liquid culture were taken in 1 ml aliquots and OD_600 nm_ measured. 3 ml gas samples were removed from the headspace of the cultures and stored in pre-evacuated 3 ml Exetainer® vials. A 50 μl gas sample was injected into a Clarus 500 gas chromatograph (PerkinElmer) with an Elite-PLOT Q (30 m × 0.53 mm internal diameter) and an electron capture detector. Carrier gas was N_2_, make-up gas was 95% (v/v) argon, 5% (v/v) methane. Standards containing N_2_O at 0.4, 5, 100, 1000, 5000, and 10 000 ppm (Scientific and Technical Gases) were measured and total N_2_O was determined as previously described.^22^

### Purification and characterisation of affinity-tagged N_2_OR from *P. denitrificans* strains

N_2_OR (NosZ) was expressed *in trans* in *P. denitrificans* using pLMB511, a derivative plasmid of the taurine-inducible expression vector pLMB509 for α-proteobacteria (Table S1†).^39^ The *Eco*RI site at position 1107 bps in pLMB509 was removed by PCR-based site-directed mutagenesis to generate pMSL001 (see Table S4† for primers). Subsequently, pMSL001 was modified by cloning of an *Nde*I–*Eco*RI fragment (Table S1† for sequence) to yield pLMB511, which has a unique *Nde*I–*Bam*HI–*Xma*I–*Eco*RI multiple cloning site that also contains the Strep-II tag sequence. As high-GC content precluded PCR gene amplification, the coding sequence of Pden_4219 (*nosZ*) was synthesised (GenScript) and cloned into pLMB511 as a *Nde*I–*Xma*I fragment, yielding pMSL002 from which N_2_OR (NosZ) with a C-terminal Strep-tag II was overproduced. The pMSL002 plasmid with Gen^R^ (20 μl ml^–1^) was conjugated into *Pd*Δ*nosZ* (PD2303), *Pd*Δ*nosL* (PD2501) and *Pd*Δ*nosZL* (PD2505) using the *E. coli* pRK2013 helper strain. Conjugants were screened for both Gen^R^/Spec^R^ and first cultured in LB and subsequently in 4 L minimal media supplemented with 2 ml l^–1^ trace element solution, at 30 °C. Overproduction of strep-tagged N_2_OR was initiated by the addition of 10 mM taurine when the culture reached OD_600 nm_ ∼ 0.6 and cultures were incubated at 30 °C for 24 h. Cells were harvested by centrifugation at 5000 × *g* and resuspended in binding buffer (20 mM HEPES, 150 mM NaCl, pH 7.2) with a protease inhibitor (cOmplete™ from Roche, 1 tablet per 50 ml resuspended cells) and lysed using a French pressure cell at 1000 psi. The cell lysate was centrifuged at 205 000 × *g* for 1 h at 4 °C and the supernatant applied to a Hi-Trap HP Strep II affinity column (5 ml, GE Healthcare). N_2_OR-Strep-tag II was eluted using elution buffer (20 mM HEPES, 150 mM NaCl and 2.5 mM desthiobiotin, pH 7.2) and exchanged back into binding buffer using a 30 kDa MWCO Centricon filter unit. Purity of the sample was confirmed using SDS-PAGE analysis and LC-MS. Protein concentrations were determined using the Bradford assay (BioRad)^40^ and bovine serum albumin as a protein standard.

UV-visible absorbance spectra of N_2_OR-Strep-tag II were recorded on a Jasco V-550 spectrophotometer. Circular dichroism spectra were recorded using Jasco J-810 Spectropolarimeter. Samples were made anaerobic by sparging with nitrogen gas for 5 min and oxidised or reduced with 5 mg ml^–1^ stocks of potassium ferricyanide and sodium dithionite, respectively, in 20 mM HEPES, 150 mM NaCl, pH 7.2, by titrating concentration equivalents. Total copper content of the protein was determined using a colorimetric bathocuproinedisulfonic acid (BCS) assay. A 100 μl protein sample was heated to 95 °C for 1 h with an equal volume of 20% (v/v) HNO_3_. The reaction was cooled and neutralised using 0.6 ml saturated ammonium sulfate solution. Copper was reduced using 100 μl hydroxylamine (100 mM) and 100 μL BCS (10 mM) added. The absorbance at 483 nm was recorded after 30 min. A standard curve was produced using a standard copper sulfate solutions (Sigma).

Activities of N_2_OR-Strep-tag II enzymes was determined using an adapted methyl viologen assay^25^,^41^ in which samples were incubated with a 500-fold excess of reduced methyl viologen. Reaction was initiated by adding N_2_O saturated buffer and the oxidation of blue (reduced) methyl viologen to its oxidised colourless form was followed at 600 nm as a function of time and data converted to specific activity using *ε*_600 nm_ = 13 600 M^–1^ cm^–1^ for the reduced methyl viologen cation radical.^41^

### Purification and characterisation of NosL

A codon-optimised gene encoding an N-terminally truncated version of *Pd*NosL (Pden_4215) was synthesised (Genscript) and sub-cloned into pET-21a(+) using 5′ *Nde*I and 3′ *Eco*RI restriction sites to generate pSPBN3. The truncation, which resulted in the replacement of the first 16 residues with a Met, was designed to simplify the expression and maturation of the protein in *E. coli* as it yielded a soluble protein located in the cytoplasmic fraction. The pSPBN3 plasmid was used to transform *E. coli* BL21 (DE3) to ampicillin (100 μg ml^–1^) resistance. Typically, 2 L flasks containing 500 ml LB supplemented with 100 μg ml^–1^ ampicillin were inoculated with 1% (v/v) of an overnight culture and grown for 2 h, 180 rpm, 37 °C until OD_600 nm_ reached ∼0.6. Expression of the NosL-encoding gene was induced by addition of 500 μM IPTG and cultures were subsequently incubated at 37 °C, 180 rpm for 5 h. Cells were harvested by centrifugation at 4000 × *g* for 15 min at 4 °C, resuspended with buffer A (50 mM MES, pH 6.5) and lysed by three rounds of sonication, each for 8 min 20 s (0.2 s intervals, 50% power), on ice. The cell lysate was centrifuged at 40 000 × *g* for 45 min at 4 °C and the supernatant applied to a DEAE column (HiPrep DEAE FF 16/10; GE Healthcare) equilibrated in buffer A. NosL was eluted using a 0–50% gradient of buffer B (50 mM MES, 1 M NaCl, pH 6.5). Fractions containing NosL were buffer exchanged using a 10 kDa MWCO Centricon into buffer A and applied to a Q-sepharose column (HiPrep Q FF 16/10; GE Healthcare) and eluted using a 20–50% gradient of buffer B. NosL-containing fractions (as determined by SDS-PAGE) were pooled and subsequently applied to an S-100 gel filtration column (120 ml, GE Healthcare) equilibrated in 100 mM MOPS, 100 mM NaCl, pH 7.5 (buffer C) and eluted in the same buffer. Fractions containing pure NosL were combined and dialysed overnight against buffer C containing 1 mM EDTA at 4 °C, and subsequently back into buffer C.

An extinction coefficient at 280 nm of 11 923 ± 5.2 M^–1^ cm^–1^, determined using a guanidine hydrochloride assay,^42^ was used to quantify the NosL protein concentration. Copper analysis revealed that the protein was purified in a copper-free form. Copper titrations were carried out using a 1 M stock solution of Cu(i)Cl (in 1 M NaCl and 0.1 M HCl) or CuCl_2_ dissolved in water. The protein was titrated with copper to give increases in the ratio of Cu : NosL of 0.1 per addition and spectra were recorded between 240 and 600 nm after each addition. Competition assays between Cu(i)-NosL and BCS were carried out to measure the dissociation constant, *K*_d_, for Cu(i)-binding to NosL, using the extinction coefficient *ε*_483nm_ = 13 300 M^–1^ cm^–1^ to determine the concentration of [CuBCS_2_]^3–^, as previously described,^33^ with 10 min incubation after each addition. Absorbance spectra were recorded using a Jasco V550 spectrophotometer.

LC-MS was conducted using a Bruker microQTof-QIII electrospray ionisation time of flight (TOF) mass spectrometer calibrated in the *m*/*z* range 300–2000 using ESI-L Low Concentration Tuning Mix (Agilent Technologies). Samples were prepared by ten-fold dilution of 50 μM protein solution with 2% (v/v) acetonitrile and 0.1% (v/v) formic acid to 0.5 ml. Samples were loaded into the LC-MS *via* an autosampler using an UltiMate 3000 HPLC system (Dionex). A 20 μl injection volume of the protein was applied to a ProSwift reversed phase RP-1S column (4.6 × 50 mm; Dionex) at 25 °C. A gradient elution was performed at a flow rate of 200 ml min^–1^ using solvents A (0.1% formic acid) and B (acetonitrile, 0.1% formic acid). Once loaded the following chromatographic method was used: isocratic wash (2% B, 0–2 min), linear gradient from 2–100% B (2–12 min), followed by an isocratic wash (100% B, 12–14 min) and column re-equilibration (2% B, 14–15 min). Mass spectra were acquired throughout using the following parameters: dry gas flow 8 l min^–1^, nebuliser gas pressure 0.8 bar, dry gas 240 °C, capillary voltage 4500 V, offset 500 V, collision RF 650 Vpp. Mass spectra from manually chosen elution volumes were averaged and deconvoluted using a maximum entropy deconvolution algorithm in Compass DataAnalysis version 4.1 (Bruker Daltonik).

Samples for non-denaturing ESI-MS were prepared in volatile buffer, ammonium acetate, 50 mM, pH 7.8. Lines were washed with anaerobic buffer prior to sample loading to ensure all O_2_ was removed and protein samples were loaded into a Hamilton syringe and directly infused into the ESI source at a rate of 300 μl h^–1^. Data was acquired in 5 min increments with ion scans between 500 and 3000 *m*/*z*. NosL mass spectra (*m*/*z* 1000–3000) were recorded with acquisition controlled by Bruker qTOF Control software, with parameters as follows: dry gas flow 4 l min^–1^, nebulise gas pressure 0.8 Bar, dry gas 180 °C, capillary voltage 4000 V, offset 500 V, quadrupole voltage 5 V, collision RF 1000 Vpp, collision cell voltage 20 V. Spectra were deconvoluted as above. Exact masses are reported from peak centroids representing the isotope average neutral mass. Predicted masses are given as the isotope average of the neutral protein or protein complex, in which Cu(i)-binding is expected to be charge compensated.^43^

Continuous wave X-band electronic paramagnetic resonance (EPR) measurements were recorded using a Bruker EMX EPR Spectrometer equipped with an ESR-900 liquid helium flow cryostat (Oxford Instruments). Spectra were recorded at 10 K with the following instrumental settings: microwave frequency, 9.4652 GHz; microwave power, 3.18 mW; modulation frequency, 100 kHz; modulation amplitude, 5 G; time constant, 82 ms; scan rate, 22.6 G s^–1^; single scan per spectrum. A 98 μM Cu(ii)–EDTA standard was used to estimate Cu(ii) concentrations for protein samples by spin integration of signal area for spectra *versus* that of the standard, where all spectra were recorded under non-saturating power conditions.

## Author contributions

S. P. B., D. J. R., A. J. G. and N. L. B. conceived the study. S. P. B., M. J. S. L., J. M. B. and D. A. S. performed the experiments. S. P. B., D. A. S., A. J. G. and N. L. B. analysed the data. S. P. B., D. J. R., A. J. G. and N. L. B. wrote the manuscript.

## Conflicts of interest

The authors declare no competing financial interests.

## Supplementary Material

Supplementary information
