# Supplementary material for: NosL is a dedicated copper chaperone for assembly of the CuZ center of nitrous oxide reductase
Source: Chem Sci. 2019 Apr 18;10(19):4985–93. doi: 10.1039/c9sc01053j (PMC6530538; doi:10.1039/c9sc01053j)
Supplement: Supplementary file 1 [file SC-010-C9SC01053J-s001.pdf]

Electronic Supplementary Material (ESI) for Chemical Science.
This journal is © The Royal Society of Chemistry 2019

Electronic Supporting Information

NosL is a dedicated copper chaperone for assembly of the Cu_Z center of nitrous oxide reductase

Sophie P. Bennett, Manuel J. Soriano-Laguna, Justin M. Bradley, Dimitri A. Svistunenko, David J. Richardson, Andrew J. Gates, Nick E. Le Brun

Supporting Tables

Table S1. Strains and plasmids used in this study.

**Name**	**Relevant Characteristics**	**Source**
Bacterial strains		
P. denitrificans PD1222	Wild-type strain, $spec^R$	1
P. denitrificans PD2303	Unmarked Δ*nosZ* deletion mutant, $spec^R$	2
P. denitrificans PD2501	Unmarked Δ*nosL* deletion mutant, $spec^R$	This study
P. denitrificans PD2505	Unmarked Δ*nosZL* double deletion mutant, $spec^R$	This study
E. coli JM101	Used as host for pK18*mobsacB*-based plasmids	3
E. coli DH5α	Used as host for plasmid modification/propagation	4
E. coli BL21 (DE3)	Used as host for recombinant protein expression from pET-based constructs	5
Plasmids		
pRK2013	Used as mobilizing plasmid in triparental crosses, kan^R	6
pK18*mobsacB*	Allelic exchange suicide plasmid, sucrose-sensitive, mob^+, kan^R	7
pSPBN1	pK18*mobsacB*-derivative, construct for *nosL* deletion, kan^R (Table S4)	This study
pMSL001	pLMB509-derivative, deficient in *Eco*RI site at 1107 bps generated by PCR using primer pair pLMB509_F1/R1 (see Table S4), *tauP*, mob^+, gen^R	This study
pLMB511	Derivative of pMSL001 with a unique *Nde*I-*Bam*HI-*Xma*I-*Eco*RI multicloning site (CATATGTGGAGCCACCCCCAATTTGAAAAAT CGAAGGGCGGGGATCCCCCGGGGACGACGACGACAA GTGGAGCCACCCCCAATTTAAAAATAAGAATTC) for recombinant strep-II tagged protein expression, *tauP*, mob$^+$, gen^R	This study
pMSL002	pLMB511 expression construct for strep-II tagged *P. denitrificans* N_2OR (NosZ; Pden_4219 sequence), gen^R	This study
pSPBN2	Complementation plasmid for *nosL* from *P. denitrificans* (Pden_4215 sequence cloned into pLMB511 as a *Nde*I-*Eco*RI fragment), gen^R	This study
pSPBN3	pET21a(+)-based expression construct for soluble NosL from *P. denitrificans* in *E. coli* BL21 (DE3), amp^R	This study

Table S2. Concentration of Cu(II) determined by double integration of S = ½ EPR signal from spectra reported in Fig. S7.

**Sample**	**$[Protein]_T$ / µM**	**$[Cu]_T$ / µM**	**$[EDTA]_T$ / µM**	**[Cu(II)] detected by EPR / µM**	**Cu(II) atoms/NosL, %**
Apo-NosL	195	-	-	1.2	0.6
Cu(I)-NosL	185	186	-	7.7	4.2
Cu(I)-NosL, EDTA	186	186	200	14.9	8.1
Cu(II)-NosL	186	187	-	14.7	7.9
Cu(II)-NosL, EDTA	186	187	201	20.6	11.1

Table S3. Determination of K_d for Cu(I) binding to NosL. Representative data are shown in Fig. S8.

**$[P]_t$ (μM)**	15.3	14.8	13.6
**$[BSC]_t$ (μM)**	160	320	800
**$[Cu]_t$ (μM)**	15.3	14.8	13.6
**$[Cu(BCS)_2{}^{3-}]$ (μM)**	7.9	9.8	12.4
**Ө, Cu occupancy**	0.483	0.33	0.088
**Cu_f (10^{-18} M)**	6.02	1.72	0.32
**K_d (10^{-18} M)**	6.45	3.49	3.38
**Average K_d (10^{-18} M)**	4.44 ± (1.73)		

Table S4. DNA sequences used in this work.

**Sequence information**	**Sequence**	
Primers for production of pMSL001	pLMB509_F1 tgccagggtcgaccaactga<br>pLMB509_R1 tcagttggtcgaccctggca	
nosL flanking regions[a] cloned into pK18*mobsacB* to produce pSPBN1	GAGAATTCacctcgctggccgtctatctggtgccgctgctggcgc<br>tgctgatgagcttcgacgccgtcgccggcgaggtcgagcgcggca<br>cgctgcccctgcttctgacctatcccgtcgcacgggccgaagttc<br>tggcgggcaagctggtcgcgcatatggcgatcctggcgctggcgg<br>tcggcgcgggctacggcgccgccgcgctggcggcggtctggaccg<br>atccggcctcgacggccgggctgccggcgctgtggcggctgatgt<br>ggagctccaccctgctgggcgcgaccttcctgggcgcgggctacg<br>cgctttccagcatcgcgcggcggccctcgggcgcggcgggactgg<br>cggtcgggctgtggctggggctggtggtgctttacgacctgggcc<br>tgctggcgctgatcgtcgccgatggcggcggcggcttcaccaccg<br>aggtgctgccggtcgcgctgcttgcgaaccctgccgacgccttcc<br>gcctgttcaacctctcggccgcccaggccaccgctgccgctgccg<br>gcgtgggcggggccgccgcgaccatcccgctttggcaatcggctc<br>tgtcggtcctggtctggccgctggccgcgcttggccttgccatcg<br>ccgcattccgaaaggtcacgccatgagacatTCTAGAtgactgac<br>tgaccgatttcctgaaccgcctgcgcgcgctgagccaacccaagg<br>agcccgcgacatgagcctgtcccgccgtcgctttctgaccatcac<br>cgccgccgtcgcgctgctgcccgcggggctgcgcgcccagcccgg<br>caggcattgggtcgggcaggcgcttggcgcccgcgcctcgatccg<br>catcgaccaccccgaggccggggcgatcaccgcccgctgcctggc<br>cgagatcgaccggctggaaaacatcctcagcctctatcgccccgg<br>cagcgcgcttgcgcgcctgaaccgcgacggcgtgctggatgcgcc<br>gccctttgaattgctcgactgcctgtcgctggccggcgccgtgca<br>tcgcgccagcggtgggctgttcgacccgaccgtgcagccgctctg<br>gtcgctctgggccgaggccgccgtgaatggccgccgtcccacgcc<br>cgaggagcggcgcgatgcgctggcacggacgggctgggatcgggt<br>gcggctggacgcggcgcgcatcacgctggagccggggatggccct<br>gacgctgaacggcatcgggcagggctatgtcgccgaccgtgtcgc<br>cgcgctgcttgaggccgagggctgggcgacattctgatcgatac<br>cggcgaattgcgggccctgggcggccggcccgatgggcgcgactg<br>gccggtccggctggccgagggcggcgcggCTGCAGTC	

[a] The sequence highlighted in yellow (a marker *Xba*I site followed by stop codons) separates the *nosL* 5'- and 3'-flanking regions. *Eco*RI and *Pst*I sites used for cloning are in capitals and underlined. Sequences in red indicate the primers used to confirm the *nosL* deletion in *P. denitrificans* PD1222.

Supporting Figures

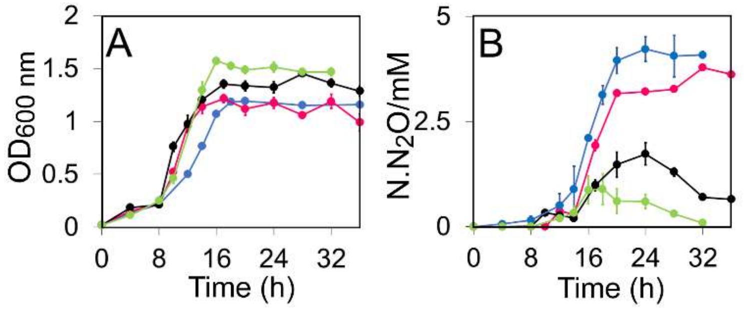

Figure S1. Growth and N_2O production characteristics of the mutant Δ*nosL* complementation under anaerobic, Cu-limited conditions in batch culture. (**A**) $OD_{600\ nm}$ and (**B**) N_2O emissions asN.N_2O (mM N in the form of N_2O). The pSPBN2 plasmid (Table S1) was conjugated into the *ΔnosL* PD2501 strain and cultured in the absence of taurine (pink circles) and presence of 1 mM taurine (black circles). For reference, data for the *ΔnosZ* PD2303 strain (blue circles) and wild type PD1222 (green circles) are shown. Bars represent SE.

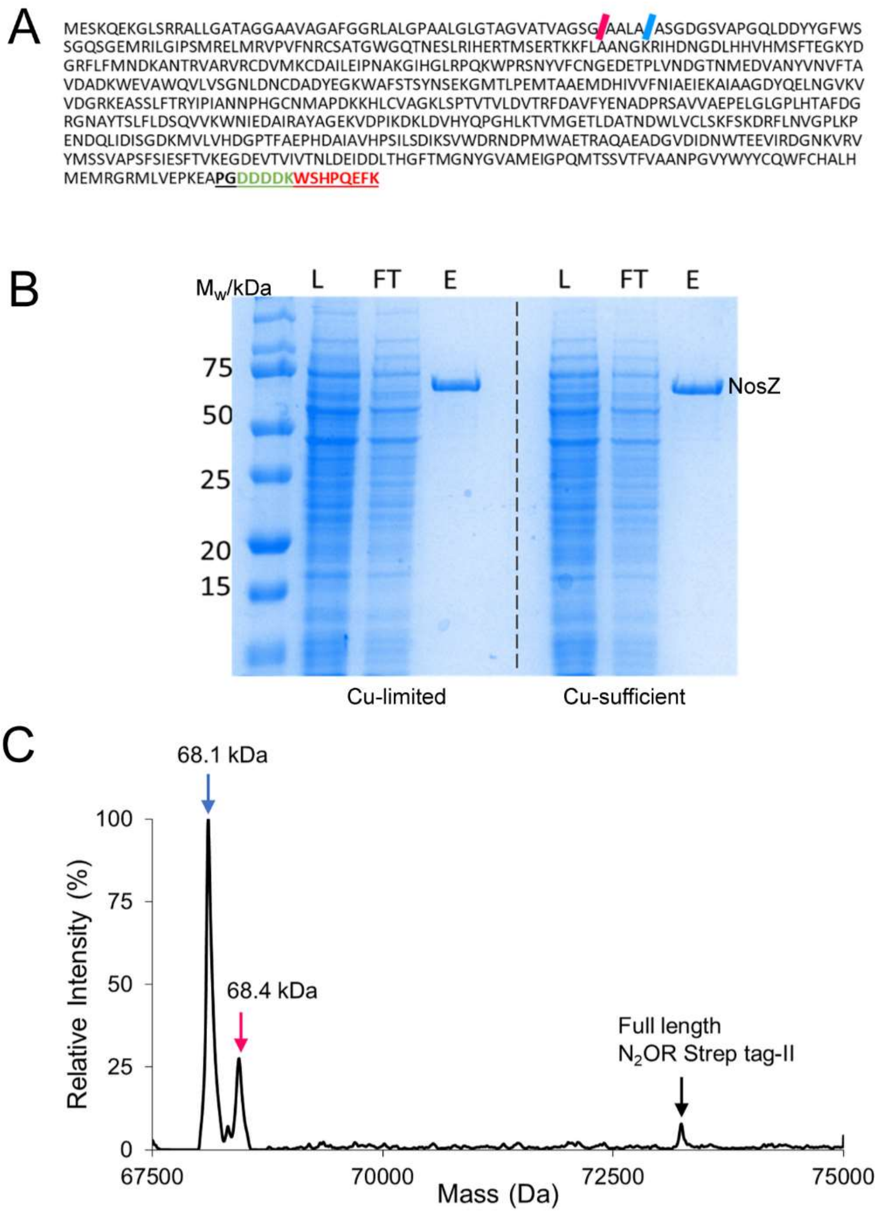

Figure S2. Analysis of the purification of N_2OR (NosZ) Strep-tag II from a wild type background. (**A**) Amino acid residue sequence generated from pMSL002 (Table S1). The sequence shown includes the C-terminal Strep-tag II (red) and enterokinase (EK) cut site (green), along with two alternative cleavage sites following export into the periplasm. (**B**) SDS-PAGE analysis of the load on to the column (L), flow through (FT) and eluent (E) from StrepII column purification of N_2OR from Cu-limited (left) and Cu-sufficient (right) cultures. The N_2OR Strep-tag II with an expected mass ~70 kDa was pure in the eluted fraction. (**C**) Deconvoluted LC-MS of N_2OR (20 µM) purified from a wild type culture grown under Cu-sufficient conditions. Sample was in 2% (v/v) acetonitrile, 0.1% (v/v) formic acid. The major species was at 68,106 Da, which corresponds to the TAT-exported, N-terminally processed form of N_2OR (predicted mass 68,105 Da). There is a small amount of a slightly larger species at 68,441 Da, which is due to N-terminal processing at an alternative cleavage site. Also present is a small amount of unprocessed N_2OR at 73,239 Da (predicted mass 73,240 Da). The presence of full-length protein was expected because the protein was purified from whole cells and therefore unprocessed cytoplasmic N_2OR, not yet exported to the periplasm, would also have been present.

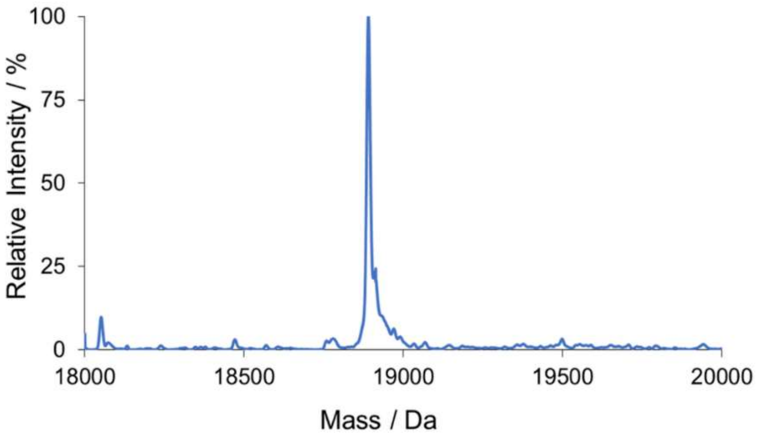

Figure S3. Deconvoluted LC-MS spectrum of purified NosL. NosL (5 μM) in 2% (v/v) acetonitrile, 0.1% (v/v) formic acid was loaded onto a reverse phase column at 25 ˚C and eluted by increasing acetonitrile concentration. A single major peak was observed, giving a mass of 18,890 Da, which compared well to the predicted mass of 18,891 Da.

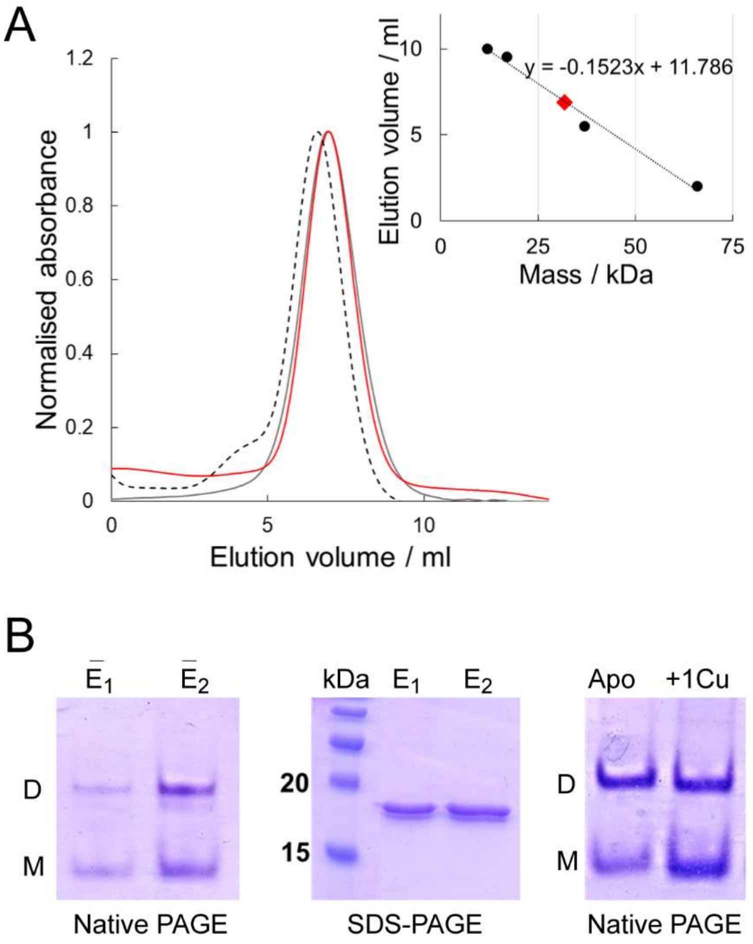

Figure S4. Association state of NosL. (**A**) Elution of apo-NosL (grey line), 1 Cu/NosL (red line) and 2 Cu/NosL (dashed black line) from an analytical gel filtration column (Generon) run using 100 mM MOPS, 100 mM NaCl, pH7.5. Inset is a gel filtration column calibration curve generated using cytochrome *c* (12.4 Da), myoglobin (17.6 Da), RsrR (35 Da) [8] and bovine serum albumin (66.5 kDa). Each volume corresponding to the elution with maximum absorbance at 280 nm is also plotted on the calibration curve: apo-NosL (orange triangle), 1Cu/NosL (blue cross) and 2 Cu/NosL (red diamond), all of which overlay. In each case, a mass of ~32 kDa was obtained, which is intermediate between a monomer and a dimer. (**B**) PAGE analysis of NosL. Left hand panel shows native PAGE analysis of two elution fractions (E1 and E2) from the main elution peak of the gel filtration experiment in (A). Middle panel shows SDS-PAGE analysis of the same fractions, showing that only NosL is present. Right panel shows native PAGE analysis of apo- and Cu-containing NosL. NosL in 100 mM MOPS, 100 mM NaCl pH 7.5 was exchanged into 60 mM HEPES, 40 mM NaCl, pH 7.2, and run on a 12% Native PAGE gel.

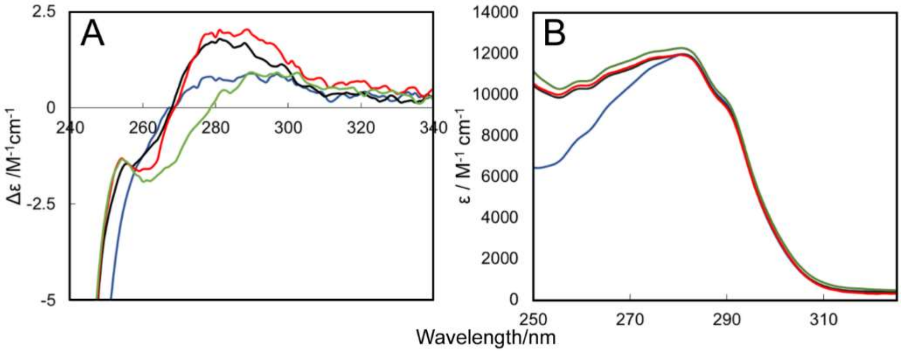

Figure S5. Copper-binding to NosL. (**A**) CD and (**B**) UV-visible absorbance spectra of apo-NosL (blue line), NosL containing 1 (black line) or 2 (green line) Cu(I) ions per protein. NosL (14.5 μM) was in 100 mM MOPS, 100 mM NaCl, pH 7.5. The 2 Cu(I)/protein sample was then passed down a desalting column equilibrated in the same buffer and the spectrum re-measured (red line). Addition of 2 Cu(I) per protein resulted in a significant change in the CD spectrum (A), with a distinct negative band at 260 nm. However, no significant differences between the absorbance spectra of NosL containing 1 and 2 Cu(I) per protein were observed (B), indicating that the second Cu(I) does not itself give rise to an absorbance. This suggests that binding of the second Cu(I) affects the existing Cu(I) site. Passage of NosL containing 2 Cu(I) per protein down a gel filtration column and re-measurement of the CD resulted in a spectrum that closely resembled that for NosL containing 1 Cu(I) per protein (A). Thus, although NosL can apparently interact with more than one Cu(I) ions, it binds only one sufficiently tightly to survive gel filtration chromatography.

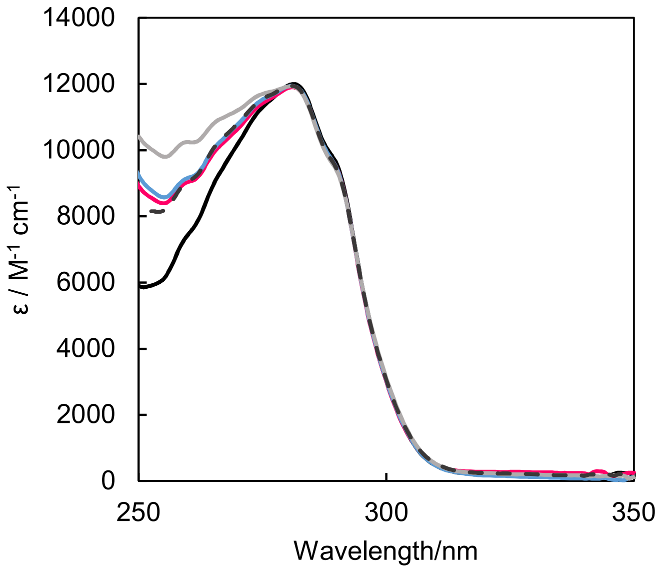

Figure S6. UV-visible absorbance spectra of apo-NosL (black line), and NosL following addition of 1 Cu(II)/protein (pink), 2 Cu(II)/protein (blue line), 0.5 Cu(I)/protein (black dash line) and 1 Cu(I)/protein (grey line). NosL (16 μM) was in 100 mM MOPS, 100 mM NaCl, pH 7.5. Path length 1 cm.

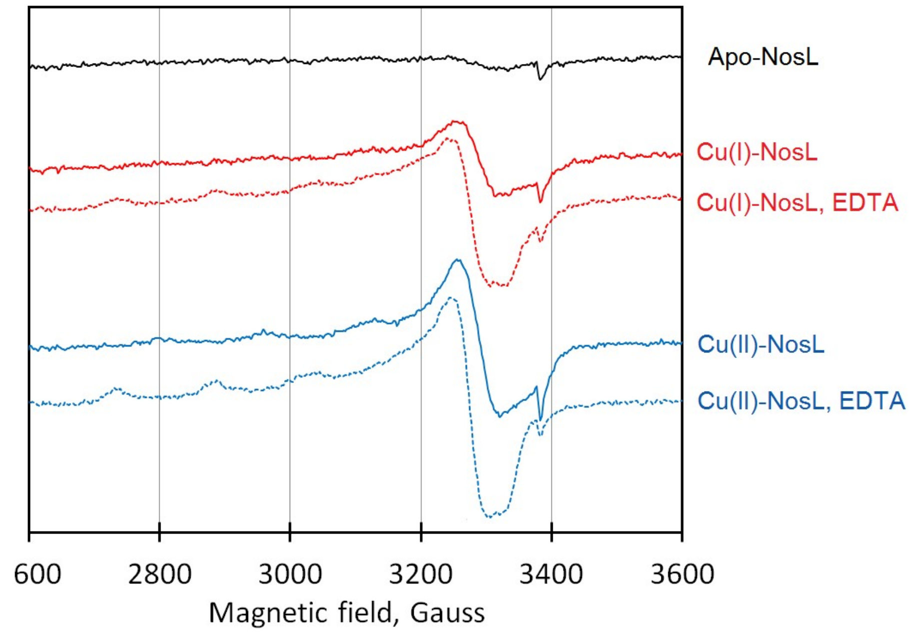

Figure S7. X-band EPR spectra of NosL samples, as indicated. The observed signals, due to S = ½ Cu(II), were quantified by comparison to a Cu(II)/EDTA standard, see Table S2. All spectra were measured at 10 K. NosL was in 100 mM MOPS, 100 mM NaCl, pH 7.5.

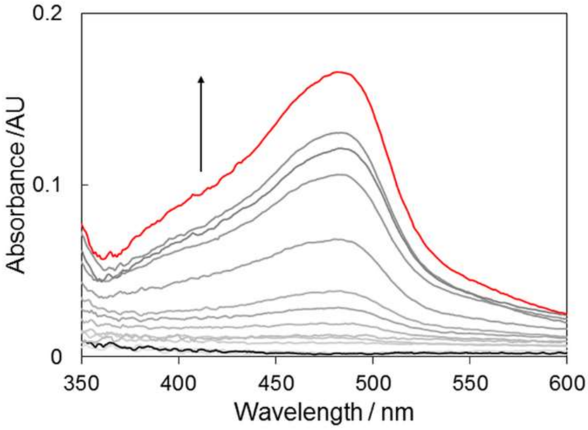

Figure S8. Determination of the Cu(I)-NosL dissociation constant using Cu(I) competition experiments. UV-visible absorbance spectra of Cu(I)-NosL with increasing amounts of the Cu(I)-chelator BCS, up to 0.8 mM. Spectra correspond to Cu(I)-NosL in the absence of BCS (black line) and Cu(I)-NosL containing a 50-fold excess of BCS (red line). A ε_{483nm} value of 13,300 $M^{-1}cm^{-1}$ was used to calculate $[Cu(I)BCS_2]^{3-}$ concentration[9]. 16 µM Cu(I)-NosL was in 100 mM MOPS, 100 mM NaCl, pH 7.5 with additions of 1 mM BCS in water.

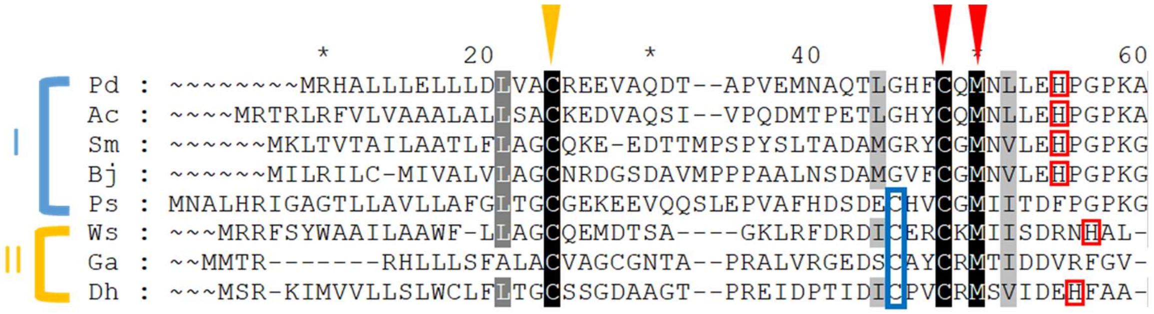

Figure S9. A comparison of part of the NosL sequence from N_2O reducing members of clade I (*Paracoccus denitrificans*-Pd*, Achromobacter cycloclastes*-Ac*, Sinorhizobium meliloti*-Sm*, Bradyrhizobium japonicum*-Bj*, Pseudomonas stutzeri*-Ps) and clade II members (*Wolinella succinogenes*-Ws*, Gemmatimonas aurantiaca T-27* -Ga *and Desulfobacterium hafniense DCB-2* -Dh). Conserved in all are the cysteine anchor (yellow triangle) and CXM motif (red triangles). Also highlighted are a histidine residue (red square) close to the CXM motif of clade I (and some clade II members), and also a second cysteine residue (blue square) among Clade II members and PsNosL. These amino acid residues could act as a third ligand to bind Cu(I), possibly resulting in different binding properties of NosL from clade I and clade II organisms.

Supporting references

1. G. E. Devries, N. Harms, J. Hoogendijk and A. H. Stouthamer, *Arch Microbiol*, 1989, **152**, 52-57.
2. M. J. Sullivan, A. J. Gates, C. Appia-Ayme, G. Rowley and D. J. Richardson, *Proc Natl Acad Sci*, 2013, **110**, 19926-19931.
3. J. Messing, *Recomb DNA Tech Bull*, 1979, **2**, 43-48.
4. D. Hanahan, *J Mol Biol*, 1983, **166**, 557-580.
5. F. W. Studier and B. A. Moffatt, *J Mol Biol*, 1986, **189**, 113-130.
6. D. H. Figurski and D. R. Helinski, *Proc Natl Acad Sci USA*, 1979, **76**, 1648-1652.
7. A. Schafer, A. Tauch, W. Jager, J. Kalinowski, G. Thierbach and A. Puhler, *Gene*, 1994, **145**, 69-73.
8. J. T. Munnoch, M. T. Martinez, D. A. Svistunenko, J. C. Crack, N. E. Le Brun and M. I. Hutchings, *Sci Rep*, 2016, **6**, 31597.
9. Z. Xiao, F. Loughlin, G. N. George, G. J. Howlett and A. G. Wedd, *J Am Chem Soc*, 2004, **126**, 3081-3090.
